# Performance improvement in polymer electrolytic membrane fuel cell based on nonlinear control strategies—A comprehensive study

**DOI:** 10.1371/journal.pone.0264205

**Published:** 2022-02-25

**Authors:** Usman Javaid, Jamshed Iqbal, Adeel Mehmood, Ali Arshad Uppal

**Affiliations:** 1 Department of Electrical and Computer Engineering, COMSATS University Islamabad, Islamabad, Pakistan; 2 Department of Computer Science and Technology, Faculty of Science and Engineering, University of Hull, Hull, United Kingdom; J.C. Bose University of Science and Technology, YMCA, INDIA, INDIA

## Abstract

A Polymer Electrolytic Membrane Fuel Cell (PEMFC) is an efficient power device for automobiles, but its efficiency and life span depend upon its air delivery system. To ensure improved performance of PEMFC, the air delivery system must ensure proper regulation of Oxygen Excess Ratio (OER). This paper proposes two nonlinear control strategies, namely Integral Sliding Mode Control (ISMC) and Fast Terminal ISMC (FTISMC). Both the controllers are designed to control the OER at a constant level under load disturbances while avoiding oxygen starvation. The derived controllers are implemented in MATLAB/ Simulink. The corresponding simulation results depict that FTISMC has faster tracking performance and lesser fluctuations due to load disturbances in output net power, stack voltage/power, error tracking, OER, and compressor motor voltage. Lesser fluctuations in these parameters ensure increased efficiency and thus extended life of a PEMFC. The results are also compared with super twisting algorithm STA to show the effectiveness of the proposed techniques. ISMC and FTISMC yield 7% and 20% improved performance as compared to STA. The proposed research finds potential applications in hydrogen-powered fuel cell electric vehicles.

## 1 Introduction

With time and exponential growth in the human population, the energy demand is increasing rapidly with the technical advancements in the electrical and electronics industries [1–4]. Energy shortfall is a serious challenge that is being faced by humans which affect social and economic developments and environment. Hence, economic and environmental susceptibility can not be guaranteed if the depletion of non-renewable energy resources continues at the present rate [5, 6].

**Fig 1:**
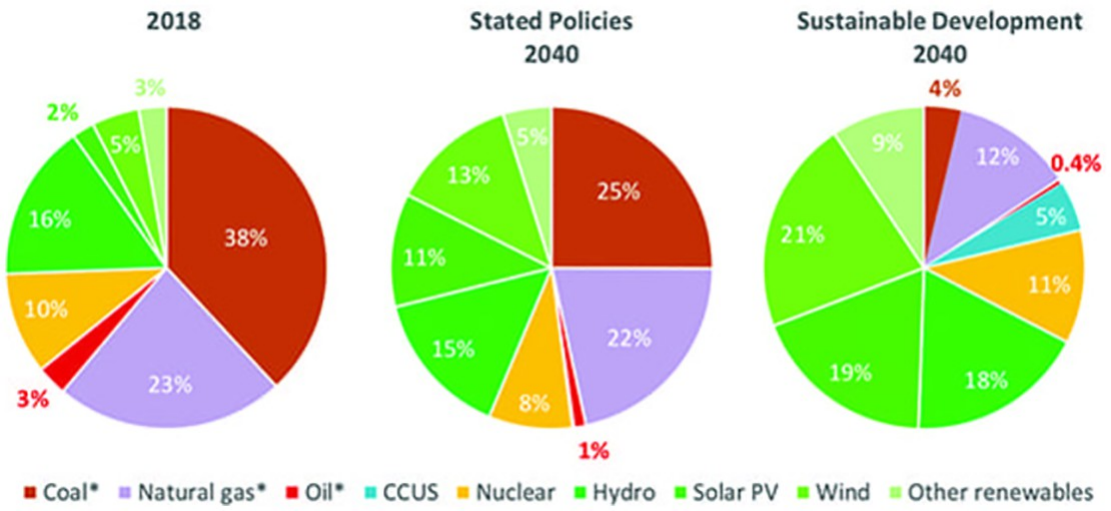
Global primary energy consumption by energy source (2010–2050) [8].

**Fig 2:**
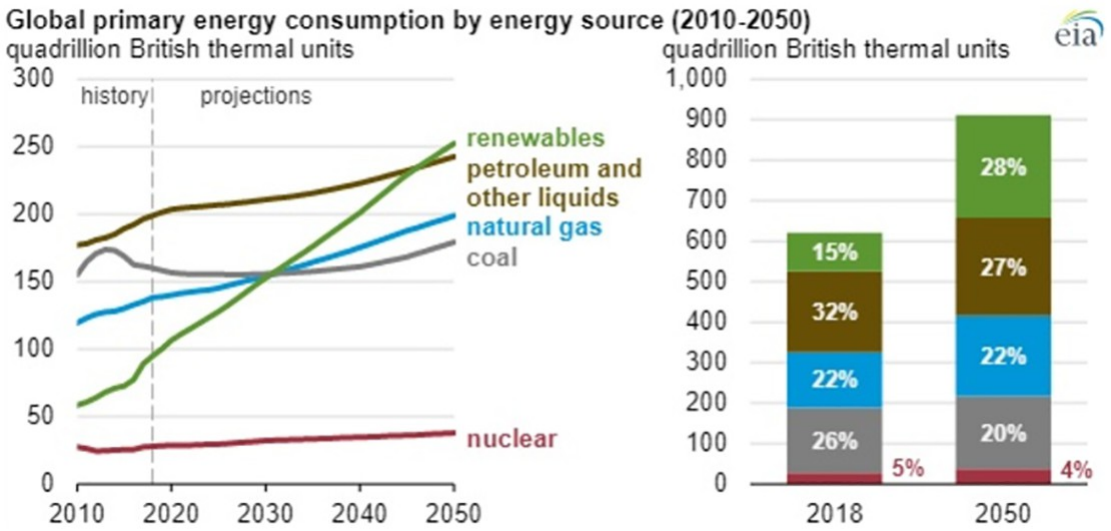
World’s energy consumption trend [9].

Various precautionary measures are insinuated to reduce the emissions of greenhouse gasses and the environmental effects associated with them. Researchers are working continuously to improve the efficiency of renewable energy sources [7]. According to Figs 1 and 2, it is indicated that the energy consumption trend will be shifted towards renewable sources in the future. The trend of renewable energy usage will surpass fossil fuels in the next 25 years [8, 9].

The world economy heavily relies on transportation which is dependent upon fossil fuels. The reduced air pollution due to lock-downs during the COVID-19 crisis indicated that a large amount of carbon and nitrogen oxides are emitted by internal combustion engines. These oxide emissions lead to severe environmental pollution [10, 11]. It is reported in [12] that our transportation system is heavily dependent on the use of fossil fuels and is responsible for producing 23% of entire nitrogen and carbon oxide emissions. Obviously, fossil fuel requirement is on the increase with the exponential growth of the human population, which heavily relies on transportation for sustainability [13]. The research community is considering various types of energy sources, including hydrogen, to overcome the threat of energy crisis faced by the human race [14]. The fossil fuel reservoirs are depleting at a speedy rate [15]. We might face an energy crisis if we do not shift our energy consumption trends towards renewable energy resources before the complete depletion of the fossil fuels [16]. The exponentially increasing population needs sustainable and reliable energy sources to meet the energy needs while considering that these sources do not negatively impact our environment [17–19]. The heat losses associated with internal combustion engines also contribute to global warming [20–22]. It is reported in [23] that parental exposure of Poly-Cyclic Aromatic Hydrocarbons (PCAH), when inhaled causes early life stress and adverse psychiatric outcomes. PCAH occurs due to the incomplete combustion of fossil fuels [24]. Therefore, special efforts are being made to reduce the use of fossil fuels. For example, small but progressive steps have been taken to ensure fossil-free operations as reported in [25].

Researchers are especially emphasizing the efficiency improvement of fuel cells by working on equilibrium stability of electrochemical reactions because this technology promises to provide a clean, sustainable and reliable renewable energy source [26]. Polymer Electrolytic Membrane Fuel Cells (PEMFCs) are getting a lot of attention because they can be used for both stationary and portable applications. PEMFCs have fast start-up time, low operating temperatures, high power density, and zero emissions, making them suitable for automobiles [27]. Some of the main advantages of fuel cells are as follows: higher efficiency of fuel cell and carbon emissions free operation as compared to internal combustion engine. The refueling of a fuel cell takes less time as compared to charging a large lead acid battery. High energy density makes them a great choice for appliances which need to be operated for a long time intervals. The disadvantages of PEM fuel cells is that reforming is used for hydrogen production which is a costly process.

There are four main subsystems in the PEMFC, which must be operated in a controlled manner to ensure smooth power generation. These four subsystems are the air delivery system, temperature management system, moisture management system and power management system.

This paper focuses on the control design of the air delivery system. In literature, numerous control techniques have been applied for controlling the air delivery system of PEMFC to overcome the effects of uncertainties and non-linearities so the efficiency of the whole system can be enhanced [28, 29]. Various control techniques have been implemented to optimise the performance of fuel cell, some of which are discussed here. An active disturbance rejection controller has been proposed in [30]. The technique uses a high-fidelity model and shows superior performance as compared to baseline controllers but the drawback of high-fidelity models is their flat frequency response. Robust adaptive control has been implemented in [31] using an approximated model using type-two fuzzy logic system. The technique has an advantage of bounded uniform error tracking, however the perceived results are based on assumption. Model reference adaptive control technique has been implemented using dynamic compressor model to overcome parameter uncertainties [32]. The limitation of the proposed technique is that the adaptive part needs to be shut after convergence. Decentralised event-triggered adaptive control has been proposed in [33]. The technique uses a reduced fourth order mathematical model and is used to overcome the nonlinear interaction problem of air supply and thermal management systems. However, the accuracy of the system is compromised by order reduction. Model-free adaptive control based on interval type-2 fuzzy logic systems has been implemented on a third order linearised plant [34]. Self-adaptive fuzzy PID has been designed using a fourth order plant in [35] which satisfies the specifications of the dynamic response, but the drawback is the difficulty in optimizing membership functions. Mamdani fuzzy method has been used as a model-free control technique to ensure reduced hydrogen consumption and improved efficiency [36]. Fuzzy predictive control using a sixth order controlled auto-regressive integrated moving average model is presented in [37] to overcome uncertainties in the system. Data-driven control has been implemented in [38], which uses original ninth order plant which was developed by Pukrushpan [39] and helps understanding system operations. First order SMC along with DC-DC converter has been presented in [40]. The technique is used to ensure fast dynamic system response and robustness against load variations. Higher-order SMC has been proposed using a second order plant to overcome the chattering phenomenon [41]. Integrated control with edge-cloud collaborative multiple tricks distributed deep deterministic policy gradient using SMC is used as a model-free technique to ensure rapid response to changing load [42]. However, the implementation of this technique requires a long-term training period. Model predictive control technique has been implemented using a linearised and reduced-order system [43]. However, linearised systems have a narrow operating range. Feedback linearisation-based multiple input multiple output model predictive control has been proposed using seventh order model along with DSP-28335 hardware in loop to ensure overshoot reduction [44]. H-infinity control is proposed using a linearised system for energy optimization of hybrid vehicles [45].

It can be observed from the above discussion that most of the control techniques have been implemented on linearised or reduced-order models. This is done to reduce the mathematical complexity of the designed controller. By reducing the system order the designed controller has limited applications. A ninth order nonlinear mathematical model of the fuel cell is considered to be the most detailed as compared to lower-order models when controlling the air delivery of PEMFC.

The nonlinear control laws have a remarkable capability to overcome uncertainties and stabilize the oxygen excess ratio (OER) of the PEMFC system and provide regulated power and voltage ratings. The ratio of the amount of oxygen provided to the fuel cell stack to the amount of oxygen used by it is known as OER. In literature higher-order SMC with STA has also been implemented but this technique ignores important model parameters. Whereas model-based control techniques are more accurate when compared to the model-free control designs. Some of the limitations of various techniques are given in the above discussion. Moreover in literature Integral Sliding Mode Control (ISMC) and Fast Terminal ISMC (FTISMC) controllers with ninth order model have not been tested to control the air-delivery system of the PEMFC.

The design of a non-linear robust controller based on ISMC and its performance comparison with FTISMC in the presence of uncertainties and transient load demands is the primary focus that has been presented in this paper. As compared to the conventional SMC technique when the system is subjected to ISMC the system will have the dimensions equal to state space and the system trajectories always tend to start from the sliding surface and hence the reaching phase is eliminated. Whereas FTISMC ensures finite-time convergence. In real-time scenarios, the instantaneous demand of reactants can increase or decrease with the fluctuations in the load driven by the fuel cell. If there is a mismatch between the supply and the demand of the reactants, the energy produced by the fuel cell will not be able to meet the actual load requirements.

**Fig 3:**
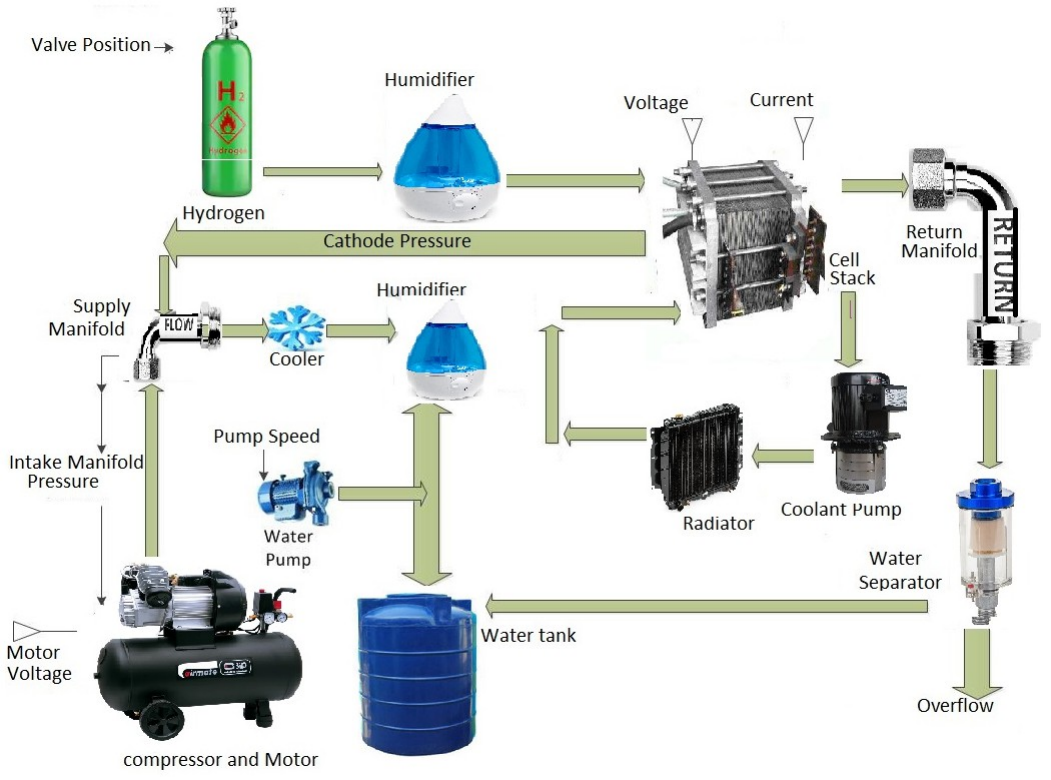
Fuel cell block diagram.

The main target is to regulate the OER of the fuel cell at a specified level. This ratio must be kept constant [46] because its fluctuation causes a drastic degradation in system performance and damages the stack structure. There will be a risk of hydrogen or oxygen starvation or excess, which may cause permanent damage to the cell stack, consequently reducing the system’s efficiency and average life span [47]. In this research work, non-linear control techniques are proposed to maintain the desired value of OER of 2 [46], so that smooth power delivery can be ensured and a constant stack voltage can be maintained for the desired load variation. A detailed block diagram of PEMFC is shown in Fig 3.

The motivation of the present research in the light of the previous work reported in [48] is summarised below:

- Work in [48] proposed a second-order SMC law based on the Super Twisting Algorithm (STA) for PEMFC. In contrast, the present work presents the design and realization of two more advanced variants of SMC i.e. ISMC and FTISMC, which have not been tested to control the air-delivery system of the PEMFC.
- For the sake of completeness, in the present research, these two variants are compared with the STA reported in [48].
- Moreover, in contrast to [48], the present work considers matched uncertainties. These uncertainties are added in the form of disturbance signal in compressor motor voltage, compressor rotational speed, and supply manifold pressure.

The main contributions of this research article are listed below:

- New applications of ISMC and FTISM have been proposed to control the air-delivery system.
- Rigorous stability analysis based on the Lyapunov criterion has been performed to evaluate the finite-time convergence of the proposed control schemes.
- Robustness of the proposed controllers has been evaluated under sudden current variations and matched uncertainties.

The rest of the article is arranged as follows: The ninth order non-linear model developed by Pukrushpan [39] is presented in Section 2. The control objective is explained in Section 3. The design of ISMC and FTISMC are presented in Section 4. The comparative analysis of various performance parameters of the above-mentioned controllers and STA in [48] is discussed in Section 5, and the manuscript is concluded in Section 6.

## 2 Control oriented model of PEMFC

After assuming that the fuel cell is operating at constant temperature and atmospheric pressure, the standard ninth order system that Pukrushpan developed can be defined by the following set of non-linear state-space equations.

$${\dot{x}}_{1}=a_{1}F_{1}F_{2}F_{5}-[\left(F_{3}\right){\left(F_{3}+\left(1-F_{3}\right)\times M_{N_{2}}\right)}^{-1}F_{4}[F_{6}{]}^{-1}]-a_{11}I_{st}$$ (1)

$${\dot{x}}_{2}={\left(1+\frac{a_{12}a_{13}}{a_{15}x_{2}+a_{14}m_{v,an}}\right)}^{-1}\left[k_{1}k_{2}x_{5}-k_{1}a_{15}x_{2}-k_{1}a_{14}m_{v,an}\right]-a_{16}I_{st}$$ (2)

$${\dot{x}}_{3}=\left(1-a_{1}\right)F_{1}F_{2}F_{5}-[1-[\left(F_{3}\right){\left(F_{3}+\left(1-F_{3}\right)M_{N_{2}}\right)}^{-1}]F_{4}{\left[F_{6}\right]}^{-1}]$$ (3)

$${\dot{x}}_{4}=\frac{a_{25}u^{2}}{x_{4}}-a_{26}u-a_{27}[{\left(\frac{x_{5}}{p_{atm}}\right)}^{a_{24}}-1]\times [a_{22}x_{4}-a_{22}x_{4}e^{F_{7}}]$$ (4)

$${\dot{x}}_{5}=a_{29}a_{22}x_{4}[1+\frac{1}{\eta_{cp}}[F_{8}]]-e^{F_{7}}-[\frac{e^{F_{7}}}{\eta_{cp}}F_{8}]-\frac{a_{30}x_{5}}{x_{6}k_{sm,out}}F_{2}$$ (5)

$${\dot{x}}_{6}=a_{22}x_{4}-a_{22}x_{4}e^{F_{7}}-F_{2}$$ (6)

$${\dot{x}}_{7}=\left(1-{\left(1+\frac{a_{12}a_{13}}{a_{15}x_{2}+a_{14}m_{v,an}}\right)}^{-1}\right)F_{9}-\left(a_{19}I_{st}-a_{20}\left(\lambda_{ca}-\lambda_{an}\right)\right)$$ (7)

$${\dot{x}}_{8}=\left(1-F_{1}\right)[F_{2}F_{5}-F_{4}\left(1-{\left(F_{6}\right)}^{-1}\right)$$ (8)

$${\dot{x}}_{9}=a_{31}\left[F_{4}-W_{rm,out}\right]$$ (9)

The state variables involved in the state equations are explained in Table 1 whereas the functions *F*_1_—*F*_9_ are presented in Table 2.

**Table 1:** Description of state variables.

State	Symbol	Description	Units
*x*_1_	$m_{O_{2}}$	oxygen mass inside the cell stack	*kg*
*x*_2_	$m_{N_{2}}$	Mass of nitrogen in the fuel cell stack	*kg*
*x*_3_	$m_{H_{2}}$	Mass of hydrogen in anode	*kg*
*x*_4_	*ω*_*cp*_	Compressors motor speed	*rad*/*s*
*x*_5_	*p*_*sm*_	Supply manifold pressure	*Pa*
*x*_6_	*m*_*sm*_	Total mass of the air in the supply manifold	*kg*
*x*_7_	$m_{w_{an}}$	Mass of moisture content at anode	*Kg*
*x*_8_	$m_{w_{ca}}$	Mass of water at cathode	*kg*
*x*_9_	*p*_*rm*_	Air pressure at return manifold	*Pa*

**Table 2:** Functions *F*_1_—*F*_9_ used in state space modeling.

$F_{1}=[1+\frac{a_{2}a_{4}}{x_{5}-x_{5}a_{10}+a_{3}}{]}^{-1}$	*F*_2_ = *k*_*sm*,*out*_(*x*_5_ − *a*_6_*x*_1_ − *a*_7_*x*_3_ − *a*_8_*m*_*v*,*ca*_)
$F_{3}=\frac{a_{6}M_{O_{2}}x_{1}}{a_{6}x_{1}+a_{7}x_{3}}$	*F*_4_ = *k*_*ca*,*out*_(*a*_6_*x*_1_ + *a*_7_*x*_3_ + *a*_8_*m*_*v*,*ca*_ − *x*_9_)
$F_{5}={\left(1+\frac{a_{9}a_{10}x_{5}}{x_{5}-x_{5}a_{10}}\right)}^{-1}\left(1+\frac{a_{9}a_{5}}{x_{5}-a_{10}x_{5}}\right)$	$F_{6}=1+\left(\frac{M_{v}a_{8}m_{v,ca}}{a_{6}x_{1}+a_{7}x_{3}}\right)\times {\left(F_{3}+M_{N_{2}}\left(1-F_{3}\right)\right)}^{-1}$
$F_{7}=‘\frac{a_{23}}{x_{4}^{2}}\left[{\left(\frac{x_{5}}{p_{atm}}\right)}^{a_{24}}-1\right]-\beta$	$F_{8}={\left(\frac{x_{5}}{p_{atm}}\right)}^{a_{24}}-1$
*F*_9_ = (*k*_1_*k*_2_*x*_5_ − *k*_1_*a*_15_*x*_2_ − *k*_1_*a*_14_*m*_*v*,*an*_)	

The exact values for the variables that have been used in this article are defined in [39].

The following equations describe the input, output, and performance variables;

$$\begin{array}{c} \dot{x}\;=\;f\left(x,u,w\right) \end{array}$$ (10)

$$\begin{array}{c} x\;=\;{\left[m_{O_{2}}\;\;m_{N_{2}}\;\;m_{H_{2}}\;\;\omega_{cp}\;\;p_{sm}\;\;m_{sm}\;\;m_{w_{an}}\;\;m_{w_{ca}}\;\;p_{rm}\right]}^{T} \end{array}$$

$$\begin{array}{c} u\;=\;V_{cm} \end{array}$$

$$\begin{array}{c} w\;=\;I_{st} \end{array}$$

The PEMFC system’s output comprises compressor flow, pressure at supply manifold, and stack voltage.

$$\begin{array}{c} y\;=\;[W_{cp}\;\;p_{sm}\;\;V_{st}] \end{array}$$ (11)

The stack voltage, OER, and the net power produced by the fuel cell are the performance variables.

$$\begin{array}{c} z\;=\;[P_{net}\;\;\lambda_{O_{2}}\;\;V_{st}] \end{array}$$ (12)

The variables involved in the state space equations are as follows.

$$\lambda_{m}=\{\begin{array}{cc} 0.043+17.81a_{i}-39.85a_{i}^{2}+36a_{i}^{2} & 0<a_{i}\leq 1\\ 14+1.4\left(a_{i}-1\right) & 1<a_{i}\leq 3 \end{array}$$

and also,

$$\begin{array}{c} W_{rm,out}=a_{32}x_{9}{\left(\frac{p_{atm}}{x_{9}}\right)}^{\frac{1}{2}}[\frac{2\gamma }{\gamma -1}[1-{\left(\frac{p_{atm}}{x_{9}}\right)}^{\frac{\gamma -1}{\gamma }}]{]}^{\frac{1}{2}}\;for\;\;\frac{p_{atm}}{x_{9}}>{\left(\frac{2}{\gamma +1}\right)}^{\frac{\gamma }{\gamma -1}} \end{array}$$

and

$$\begin{array}{c} W_{rm,out}=a_{32}x_{9}\gamma^{\frac{1}{2}}{\left(\frac{2}{\gamma +1}\right)}^{\frac{\gamma +1}{2\left(\gamma -1\right)}}\;for\;\frac{p_{atm}}{x_{9}}\leq {\left(\frac{2}{\gamma +1}\right)}^{\frac{\gamma }{\gamma -1}} \end{array}$$

The model parameters are given in Table 3.

**Table 3:** Coefficients defined in the fuel cell.

$a_{1}=x_{O_{2,ca,in}}$	$a_{2}=\frac{M_{v}}{M_{a,ca,in}}$	$a_{3}=\phi^{des}p_{sat,T_{cl}}-\phi_{ca,in}p_{sat,T_{cl}}$	$a_{4}=\phi_{ca,in}p_{sat,T_{cl}}$
$a_{5}=\phi^{des}p_{sat,T_{cl}}$	$a_{6}=\frac{R_{O_{2}}T_{st}}{V_{ca}}$	$a_{7}=\frac{R_{N_{2}}T_{st}}{V_{ca}}$	$a_{8}=\frac{R_{v}T_{st}}{V_{ca}}$
$a_{9}=\frac{M_{v}}{M_{a}}$	$a_{10}=\frac{\phi_{atm}p_{sat,T_{atm}}}{p_{atm}}$	$a_{11}=\frac{M_{O_{2}}n}{4F}$	$a_{12}=\frac{M_{v}}{M_{H_{2}}}$
$a_{13}=p_{v,ca,in}=\phi_{ca,in}p_{sat,T_{cl}}$	$a_{14}=\frac{R_{v}T_{st}}{V_{an}}$	$a_{15}=\frac{R_{H_{2}}T_{st}}{V_{an}}$	$a_{16}=\frac{M_{H_{2}}n}{2F}$
$a_{17}=\frac{M_{v}n}{2F}$	$a_{18}=\frac{\rho_{m,dry}}{M_{m,dry}}$	$a_{19}=\frac{n_{d}}{A_{fc}F}$	$a_{20}=\frac{D_{w}a_{18}}{t_{m}}$
*a*_21_ = *M*_*v*_*A*_*fc*_*n*	$a_{22\frac{\Phi_{max}\rho \pi d_{c}^{2}K_{u_{c}}\delta }{4\theta^{0.5}}}$	$a_{23}=\frac{\beta 2C_{p}T_{cp,in}}{K_{u_{c}}^{2}\psi_{max}}$	$a_{24}=\frac{\gamma -1}{\gamma }$
$a_{25}=\frac{\eta_{cm}}{J_{cp}R_{cm}}$	$a_{26}=\frac{\eta_{cm}k_{v}}{J_{cp}R_{cm}}$	$a_{27}=\frac{C_{p}T_{atm}}{ef_{mec}\eta_{cp}J_{cp}}$	$a_{28}=a_{23}{\left(\frac{1}{p_{atm}}\right)}^{a_{24}}$
$a_{29}=\frac{\gamma R_{a}T_{atm}}{V_{sm}}$	*a*_30_ = *k*_*sm*,*out*_*γ*	$a_{31}=\frac{R_{a}T_{rm}}{V_{rm}}$	$a_{32}=\frac{C_{D,rm}A_{T,rm}}{\sqrt{\bar{R}T_{rm}}}$

The abbreviations and subscripts are defined in the Tables 1 and 2 respectively in S1 Appendix. The model parameters are taken from [39].

## 3 Control objective

**Fig 4:**
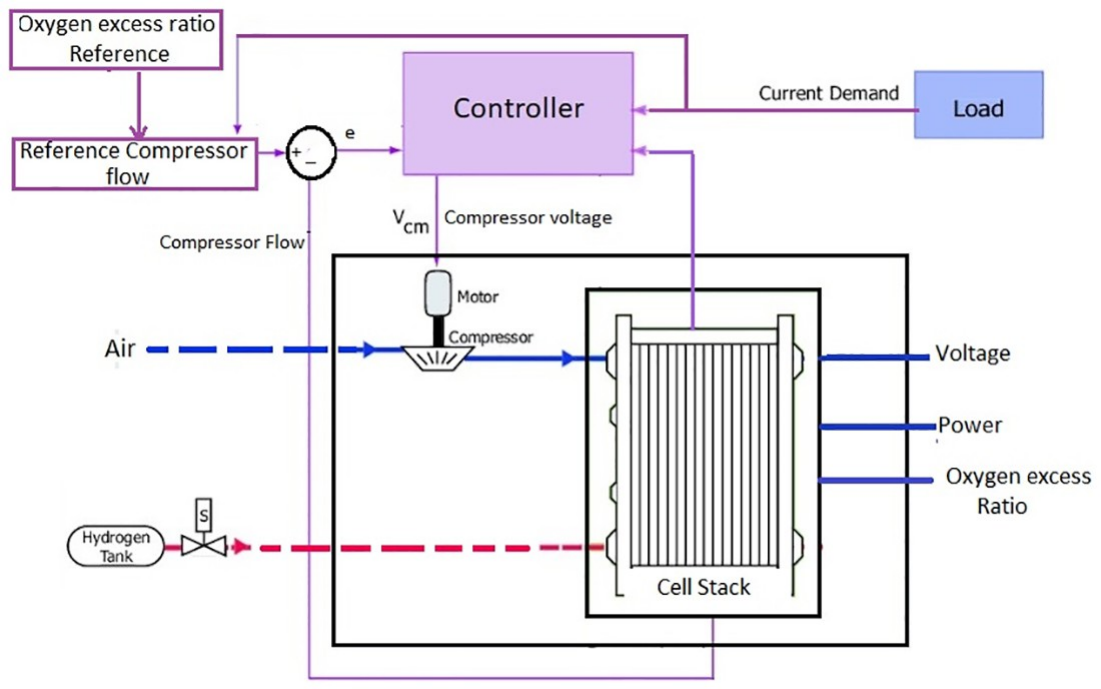
Air delivery system of PEMFC.

Although there are four main subsystems that require a proper control mechanism to achieve maximum performance efficiency of the PEMFC, this paper is particularly restricted to the control designing of the air delivery subsystem to maintain the OER at a specified level. The air delivery system for PEMFC is demonstrated in Fig 4.

The main objective is to design the control signal for the motor compressor voltage, which will, in turn, regulate the OER denoted by $\lambda_{O_{2}}$ at a specified level to obtain maximum efficiency. OER is defined mathematically as

$$\begin{array}{c} \lambda_{O_{2}}=\frac{W_{O_{2,in}}}{W_{O_{2,react}}} \end{array}$$ (13)

To fulfill the requirements of PEMFC for perfect operation, $\lambda_{O_{2}}$ should be maintained at 2 as specified in [39]. This is our control objective that can be achieved by regulating the compressor motor speed, which in turn is controlled by compressor motor voltage. The controllers’ performance has been observed under the presence of matched uncertainties which have been added in the dynamics of *ω*_*cp*_ and *p*_*sm*_.

## 4 Control design

FTISMC and ISMC have been designed to regulate the air delivery system for OER of PEMFC. The compressor speed $\omega_{c_{p}}$ is used to regulate the oxygen inlet flow because the oxygen inlet flow cannot be directly measured. So OER is controlled by controlling the compressor speed. The OER is regulated by ensuring that the error signal remains zero. The error signal is defined as

$$\begin{array}{c} e\left(x,t\right)=W_{cp}-W_{cp,ref} \end{array}$$ (14)

By using reference airflow at the cathode terminal, the mass flow reference *W*_*cp*,*ref*_ can be found from the oxygen flow reference at the cathode. The required mass flow of air can be given as

$$\begin{array}{c} W_{dry,ref}=\frac{1}{x_{O_{2}}}W_{O_{2},ca,ref}=\frac{1}{x_{O_{2}}}\lambda_{O_{2,ref}}M_{O_{2}}\frac{nI_{st}}{4F} \end{array}$$ (15)

Considering the relative humidity of the air, the required flow rate of air can be given as

$$\begin{array}{c} W_{cp,ref}=\left(1+\omega_{amb}\right)\frac{1}{x_{O_{2}}}\lambda_{O_{2,ref}}M_{O_{2}}\frac{nI_{st}}{4F} \end{array}$$ (16)

So if we substitute these equations in (14) the equation becomes

$$\begin{array}{c} e\left(x,t\right)=b_{11}\left(1-d\left(x\right)\right)x_{1}-\left(1+\omega_{amb}\right)\frac{1}{x_{O_{2}}}\lambda_{O_{2,ref}}M_{O_{2}}\frac{nI_{st}}{4F} \end{array}$$ (17)

### 4.1 Fast terminal integral sliding mode control

Due to the finite time convergence of the tracking error, FTISMC is extensively used in literature. However, singularity issues still occur. So in this paper, innovative FTISMC has been proposed to resolve the singularity issues by canceling out the effects of non-parametric and parametric disturbances. Furthermore, unlike Conventional SMC, FTISMC provides finite-time convergence, both in the reaching and sliding phases. The following first-order nonlinear differential dynamics are presented to describe the respective sliding manifold of FTISMC. The control of the air delivery system of PEMFC has been attained by using a sliding manifold as in (18)

$$\begin{array}{c} s=\alpha e\left(t\right)+\beta \int {\left|e\right|}^{\gamma }Sign\left(e\right)dt \end{array}$$ (18)

The above sliding surface is defined to obtain the desired level of $\lambda_{O_{2}}$. The term |*e*|^*γ*^ in the sliding surface ensures the finite time convergence of the error dynamics. Where *γ* = *p*/*q*, *p* and *q* are positive odd integers. Where the tracking error is defined by the variable *e* and *α*, *β*, *γ* are used as tuning parameters. While choosing the value of *α* and *β*, it must be considered that the Hurwitz condition is satisfied i-e *α* > 0 and *β* > 0, Whereas 0 < *γ* < 1. The time derivative of the sliding manifold is given by the (19)

$$\begin{array}{c} \dot{s}=\alpha \dot{e}\left(t\right)+\beta {\left|e\right|}^{\gamma }Sign\left(q\right) \end{array}$$ (19)

The strong reachability law is given as:

$$\begin{array}{c} \dot{s}=-K_{1}s-K_{2}Sign\left(s\right) \end{array}$$ (20)

The controller consists of two parts, i.e. the continuous part which is given in (26) and the discontinuous part given in (29). It is assumed that the fuel cell is operating at a fixed temperature and atmospheric pressure. The first-order derivative of the sliding manifold provides the continuous part of the controller. The error *e* is characterised as

$$\begin{array}{c} e=W_{cp}-W_{cp,ref} \end{array}$$ (21)

The time derivative of error can be written in the following form

$$\begin{array}{c} \dot{e}=\left(au^{2}+bu+c\right) \end{array}$$ (22)

where

$$a=[a_{22}-a_{22}e^{F_{7}}+\frac{2a_{22}a_{23}}{p_{atm}^{a_{24}}}\times e^{F_{7}}x_{5}^{3}x_{4}^{-2}-2a_{22}a_{23}e^{F_{7}}x_{4}^{-2}]c_{4}x_{4}^{-1}$$ (23)

$$b=-a_{26}[a_{22}-a_{22}e^{F_{7}}+\frac{2a_{22}a_{23}}{p_{atm}^{a_{24}}}\times e^{F_{7}}x_{5}^{3}x_{4}^{-2}-2a_{22}a_{23}\times e^{F_{7}}x_{4}^{-2}]$$ (24)

$$\begin{array}{cc} c & =-[a_{22}-a_{22}e^{F_{7}}+\frac{2a_{22}a_{23}}{p_{atm}^{a_{24}}}\times e^{F_{7}}x_{5}^{3}x_{4}^{-2}-2a_{22}a_{23}\times e^{F_{7}}x_{4}^{-2}]\\ & \frac{a_{27}}{k_{sm,out}}F_{2}[a_{22}x_{4}-a_{22}x_{4}\left[e^{F_{7}}\right]]-\left(\frac{a_{22}a_{23}a_{24}e^{F_{7}}x_{4}^{-1}x_{5}^{a_{24}-1}}{p_{atm}^{a_{24}}}\right)\times \\ & \;\left(a_{29}a_{22}x_{4}\left[1+\frac{1}{\eta_{cp}}F_{8}\right]\right)-e^{F_{7}}-\frac{e^{F_{7}}}{k_{sm,out}\eta_{cp}}F_{2}-a_{30}\frac{x_{5}}{x_{6}k_{sm,out}}F_{2}\\ & \;-2.8\times 10^{-2}d\frac{dI_{st}}{dt} \end{array}$$ (25)

The continuous part of FTISMC control input can be found by using the quadratic formula given as

$$\begin{array}{c} u_{eq\left(1,2\right)}=\frac{-b\pm \sqrt{b^{2}-4a\left(c+{\left(\beta /\alpha \right)|q|}^{\gamma }Sign\left(q\right)\right)}}{2a} \end{array}$$ (26)

The Eq (26) has two forms which are given in (27) and (28). During simulation ot has been found that (28) renders the system unstable. So (27) is used for the continuous part of the controller

$$\begin{array}{c} u_{eq\left(1,2\right)}=\frac{-b+\sqrt{b^{2}-4a\left(c+{\left(\beta /\alpha \right)|q|}^{\gamma }Sign\left(q\right)\right)}}{2a} \end{array}$$ (27)

$$\begin{array}{c} u_{eq\left(1,2\right)}=\frac{-b-\sqrt{b^{2}-4a\left(c+{\left(\beta /\alpha \right)|q|}^{\gamma }Sign\left(q\right)\right)}}{2a} \end{array}$$ (28)

The discontinuous control part is given by

$$\begin{array}{c} u_{dis}=-K_{1}s-K_{2}sign\left(s\right) \end{array}$$ (29)

Where *K*_1_ and *K*_2_ are tuning parameters, and their values must be greater than zero.

To reduce the chattering phenomena and singularity issues in FTISMC, saturation functions are used instead of using the simple *sign*() function in the discontinuous and as-well-as continuous parts of the controller, which is defined as follows.

$$\begin{array}{c} sat\left(s\right)=\{\begin{array}{cc} 1 & s>0\\ K_{sat}s & s|s|\leq \delta \\ -1 & sl-\delta \end{array} \end{array}$$ (30)

Where $K_{sat}=\frac{1}{\delta }$ and *δ* is called the boundary layer. The FTISMC based law is given as the sum of continuous and discontinuous control laws

$$\begin{array}{c} u=u_{eq}+u_{dis} \end{array}$$ (31)

#### 4.1.1 Existence of fast terminal integral sliding mode

The existence of FTISMC can be proved by evaluating Lyapunov stability as follows;

$$\begin{array}{ccc} V & = & \frac{1}{2}S^{2} \end{array}$$ (32)

$$\begin{array}{ccc} \dot{V} & = & S\dot{S}=S\left(au^{2}+bu+c+{\left(\beta /\alpha \right)|q|}^{\gamma }sign\left(q\right)\right) \end{array}$$ (33)

where,

$$\begin{array}{ccc} \;\;u & = & \frac{-b+\sqrt{b^{2}-4a\left(c+{\left(\beta /\alpha \right)|q|}^{\gamma }sign\left(q\right)\right)}}{2a}-K_{1}s-K_{2}sign\left(s\right)) \end{array}$$ (34)

After substituting the value of *u* in (33) we get

$$\begin{array}{ccc} \dot{V} & = & S[0-K_{1}S-K_{2}sign\left(S\right)] \end{array}$$ (35)

$$\begin{array}{ccc} \dot{V} & \leq & \left(-K_{1}S^{2}-K_{2}|S|\right) \end{array}$$ (36)

$\dot{V}\leq 0,\;\forall \;K_{1}>0,\;\forall \;K_{2}>0$ Thus proving the existence of FTISMC.

The equation for $\dot{V}$ can be rearranged as shown as

$$\begin{array}{c} \dot{V}+K_{1}S^{2}+K_{2}\left|S\right|\leq 0 \end{array}$$ (37)

Put the value of S in terms of V the equation becomes

$$\begin{array}{c} \dot{V}+K_{1}2V+K_{2}\sqrt{2V}\leq 0 \end{array}$$

Now assuming $\bar{K_{1}}=2K_{1}$ and $\bar{K_{2}}=2\sqrt{K_{2}}$ the inequality can be written as

$$\begin{array}{c} \dot{V}+\bar{K_{1}}V+\bar{K_{2}}\sqrt{V}\leq 0 \end{array}$$

The finite time required to reach the sliding manifold is given as (cf. [49])

$$T_{S}\leq \frac{1}{2{\bar{K}}_{1}}\text{ln}\left(\frac{{\bar{K}}_{1}V^{1/2}\left(S_{}\left(0\right)\right)+{\bar{K}}_{2}}{{\bar{K}}_{2}}\right)$$ (38)

The Eq (38) indicates the finite time convergence of the system. The term *β*∫|*e*|^*γ*^ *Sign*(*e*)*dt* acts as a terminal attractor and the integral term also eliminates the reaching phase of the conventional SMC. Thus ensuring smooth steady state operation along with fast transient response.

### 4.2 Integral sliding mode control

ISMC has a remarkable tendency to eliminate the reaching phase of the control system by striking sliding mode all around the system behavior and guaranteeing invariance right from the starting time [50]. The basic approach used in this technique is to include an integral term in the equation of sliding manifold. When the integral term is the part of the sliding manifold, it eliminates the reaching phase [51]. The sliding manifold function can be defined as in (39)

$$\begin{array}{c} S=c\dot{e}\left(t\right)+e\left(t\right)+\int e\left(t\right)dt \end{array}$$ (39)

where *e* is the tracking error defined in (14) and *c* is the tuning parameter [52]. The above sliding surface is designed to ensure the exponential stability of the error dynamics in the sliding mode. This can be achieved if the polynomial *cρ*^2^ + *ρ* + 1 (*ρ* is the Laplace operator) related to (39) is Hurwitz.

As the relative degree of PEMFC for controller design of the *ω*_*cp*_ is 1, so we can define the sliding manifold as in (39). The continuous control part is obtained similarly as in the previous section. But in this technique, an integral error signal is added in the equation of sliding manifold. The time derivative of the surface yields (40). The equation is in quadratic form and its solution is given in (41). Which has two parts given by (42) and (43).

$$\begin{array}{c} \dot{S}=au^{2}+bu+c+e\left(t\right) \end{array}$$ (40)

where

$$\begin{array}{c} u_{eq\left(1,2\right)}=\frac{-b\pm \sqrt{b^{2}-4a\left(c+e\left(t\right)\right)}}{2a} \end{array}$$ (41)

$$\begin{array}{c} u_{eq\left(1,2\right)}=\frac{-b+\sqrt{b^{2}-4a\left(c+e\left(t\right)\right)}}{2a} \end{array}$$ (42)

$$\begin{array}{c} u_{eq\left(1,2\right)}=\frac{-b-\sqrt{b^{2}-4a\left(c+e\left(t\right)\right)}}{2a} \end{array}$$ (43)

#### 4.2.1 Existence of integral sliding mode

The existence of ISMC can be proved by evaluating Lyapunov stability as follows;

$$\begin{array}{ccc} V & = & \frac{1}{2}S^{2}\\ \dot{V} & = & S\dot{S}=S\left(au^{2}+bu+c+e\left(t\right)\right) \end{array}$$ (44)

where,

$$\begin{array}{ccc} \;\;u & = & \frac{-b+\sqrt{b^{2}-4a\left(c+e\left(t\right)\right)}}{2a}-KSign\left(S\right) \end{array}$$ (45)

After substituting the value of *u* from (45) in $\dot{V}$ we get

$$\begin{array}{ccc} \dot{V} & = & S[0-KSign\left(S\right)] \end{array}$$ (46)

$$\begin{array}{ccc} \dot{V} & \leq & -K|S| \end{array}$$ (47)

$\dot{V}\leq 0,\;\forall \;K>0$ confirms the fast convergence of system trajectories to the sliding manifold and hence ensuring the existence of ISMC. ISMC eliminates the reaching phase by including an integral term. The equation for $\dot{V}$ can be rearranged as shown as

$$\begin{array}{c} \dot{V}+K\left|S\right|\leq 0 \end{array}$$

Put the value of S in terms of V the above equation becomes

$$\begin{array}{c} \dot{V}+K\sqrt{2V}\leq 0 \end{array}$$

Now assuming $\bar{K}=2\sqrt{K}$ the inequality can be written as

$$\begin{array}{c} \dot{V}+\bar{K}V+\leq 0 \end{array}$$

Using the results in [53], the finite time required for converging of systems’ trajectories to the sliding manifold can be characterised as

$$\begin{array}{c} \begin{array}{cc} T_{S} & \leq \left(\frac{\sqrt{2VS\left(0\right)}}{{\bar{K}}_{2}}\right) \end{array} \end{array}$$

The term *T*_*S*_ is used to calculate the finite time convergence of the system. The integral term in the sliding surface eliminates the reaching phase of the system.

In FTISMC, the error dynamics in the sliding mode converges in finite time, whereas, in ISMC the error dynamics converges exponentially.

## 5 Outcomes of research

The simulations are performed in MATLAB/Simulink. The comparison of the designed controllers, and their comparison with STA in [48] is presented in this article. In the presence of disturbances and uncertainties along with varying power requirements, the behavior of performance variables including stack voltage, power, and OER have been recorded.

**Fig 5:**
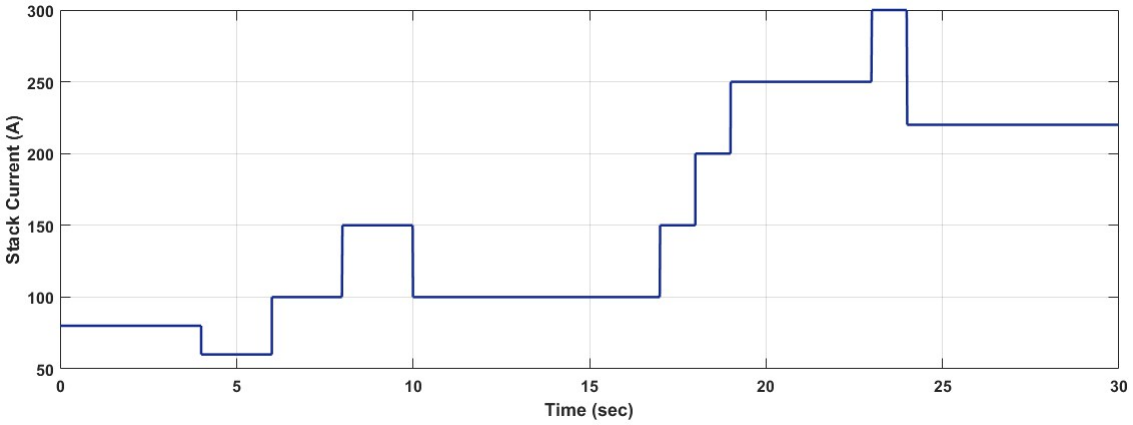
Stack current created from step functions.

A variable current signal that varies from 60A to 300A depicts the various current demands regarding changing time intervals. The current waveform, which is being considered as disturbance input for the fuel cell, is shown in Fig 5. To ensure smooth power delivery and optimum performance at all times, the compressor motor voltage must be adjusted accordingly to meet the current demands in the presence of disturbances. For the compilation of these results and the disturbances, the matched uncertainties have also been added in compressor motor voltage, compressor rotational speed, and supply manifold pressure.

### 5.1 Simulation results

**Fig 6:**
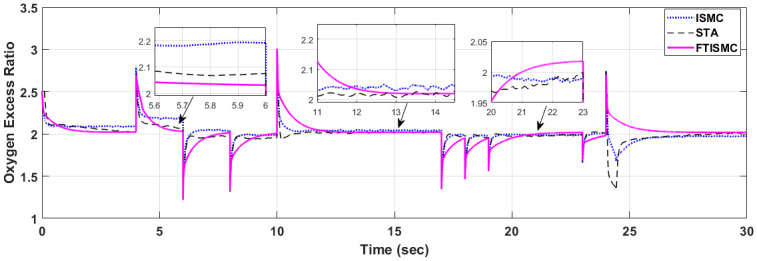
Comparison of OER for the three controllers.

The control strategies proposed in this paper are designed to maintain the OER at a specified value of 2 by controlling the compressor motor speed, which is maintained by the compressor motor voltage signal. The overshoot in the response is caused by the sudden increase in the demand current, whereas, the undershoot appears when the demand current decreases abruptly. The comparative analysis of all three controllers indicates that all the control laws ensure smooth power delivery and are capable of regulating the air delivery system by maintaining the OER at the specified level. But on closer inspection of the simulation results, it can be seen that FTISMC outperforms ISMC and STA with faster settling time and lesser overshoots. Fig 6 depicts the performance comparison of all three controllers. The comparative results indicate that FTISMC has better performance in terms of percentage overshoots in the presence of large fluctuating current demands as compared to the other two control techniques for example, at t = 24 s, the dip for STA is at 1.3, for ISMC, it is 1.6 and for FTISMC there is no overshoot and it is settling at the set value of 2. Similarly, at t = 10 s STA has an overshoot dip at 2.1, whereas there is no overshoot in the case of FTISMC and ISMC. FTISMC depicts a critically damped response plot, and the curve is settling nearest to the set value of 2, whereas the curves for ISMC ans STA have little fluctuations and thus depict steady state error. The settling time for STA and FTISMC is 1 second. The zoomed versions for the curves of OER have been generated which depict the finite time convergence of the FTISMC controller. Thus we can conclude that FTISMC ensures best steady state performance among the three controllers with faster settling times, and a smooth compressor motor voltage signal ensures smooth working and optimal fuel efficiency of the PEMFC.

**Fig 7:**
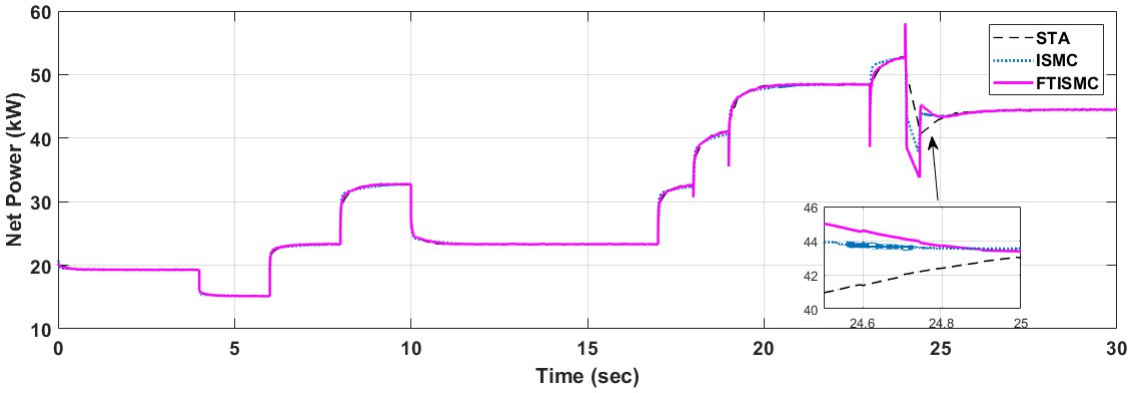
Net power produced by PEM fuel cell.

**Fig 8:**
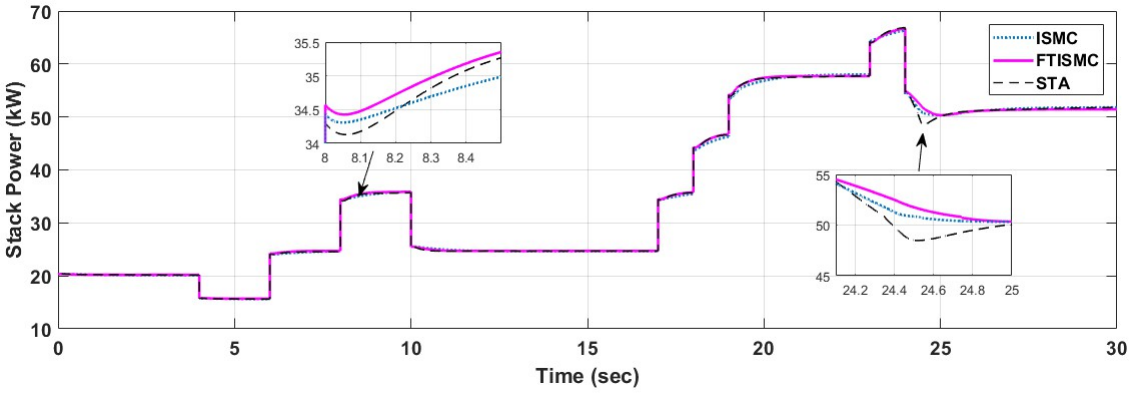
Stack power produced by PEM fuel cell.

The graph shown in Fig 7 presents the total power generated by the fuel cell stack. The compressor motor is acting as a load to the fuel cell. Thus the net power is the difference between the stack power and the power consumed by the compressor. Results show that transient response of power is improved with FTISMC, which outperforms ISMC and STA. The results indicate that at t = 24 s there is a reduction in power demand according to the stack current graph. The curve for FTISMC drops down to 34kW, followed by ISMC at 37Kw, whereas the curve for STA drops down to 41kW. Similarly, the stack power generated by the fuel cell is shown in Fig 8.

**Fig 9:**
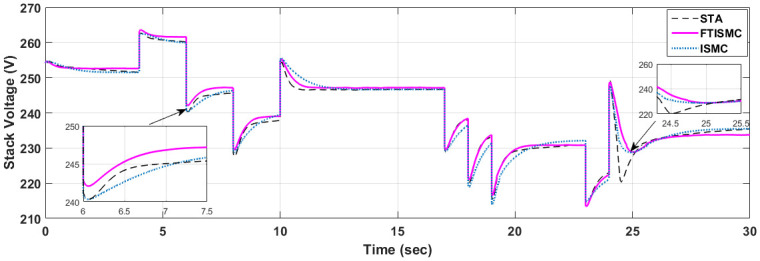
Stack voltage produced by fuel cell.

Fig 9 illustrates the comparative performance analysis of the stack voltage generated by the PEMFC system under different control laws. It can be observed that the curve for FTISMC outperforms ISMC and STA in terms of settling time. For example, the disturbances in the form of current demand at t = 24 s the largest overshoot arises in the case of STA with a peak at 220V. ISMC and FTISMC have overshoots at 229 V. Therefore, it can be concluded that FTISMC outperforms STA and ISMC when sudden variations in current demand are required in the presence of matched uncertainties. Similarly, for the disturbance in current demands at t = 10 s, STA exhibits an underdamped response with a settling time of 1.5 s. ISMC indicates an overdamped response with a settling time of 3 seconds, and FTISMC indicates a critically damped response with a settling time of 1 second. Thus ensuring lesser voltage fluctuations which would otherwise cause instability in electronic equipment under higher current variation in the presence of uncertainties. So the use of FITSMC ensures smooth and uniform voltage delivery from a PEMFC system.

**Fig 10:**
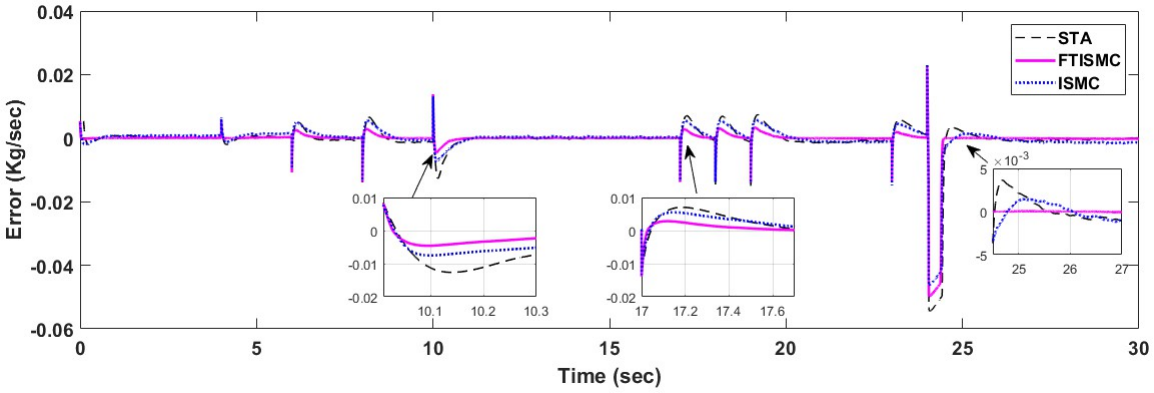
Comparison of error tracking.

To control the air delivery system for OER regulation, the value of the error signal should remain strictly close to zero *e*(*t*) → 0. The error tracking curves for all the three controllers are given in Fig 10. It can be observed that for all the disturbances of current variations, the graph for FTISMC remains close to zero, indicating the best error tracking as compared to ISMC and STA. Overshoot in error is maximum in the case of STA for all the current demand variations at times, whereas in the case of FTISMC, it is at 0.0025 percent. Moreover, if we observe the graphs at t = 24 s, the FTISMC has a quicker response followed by ISMC with an approximate delay of 2.5 s, and STA has almost the same settling time with an overshoot of 0.004 percent. The zoomed version of the error tracking curve at t = 10–10.3, 17–17.6 and 24–27 s demonstrate the effectiveness of FTISMC in terms of finite time error tracking along with finite time stability. Therefore, it can be concluded that FTISMC has better error tracking as compared to ISMC and STA in sudden current demand variations under the presence of matched uncertainties. The quantitative analysis of the error dynamics is presented in Table 4.

**Table 4:** Quantitative analysis of control schemes.

Control Scheme	*E*_*rms*_	*CE*_*rms*_	*E*_*Mean*_	*E*_*Median*_	*E*_*Range*_	*E*_*STD*_
**FTISMC**	0.0051	8.7	0.0004791	1.725*e*^−6^	0.071	0.005015
**ISMC**	0.0064	4.3	0.000827	0.0003921	0.0694	0.007064
**STA**	0.0071	1.8	0.000709	−1.284*e*^−16^	0.077	0.00632

**rms** Root mean square, **STD** Standard Daviation, **E** Error, **CE** Control Effort

**Fig 11:**
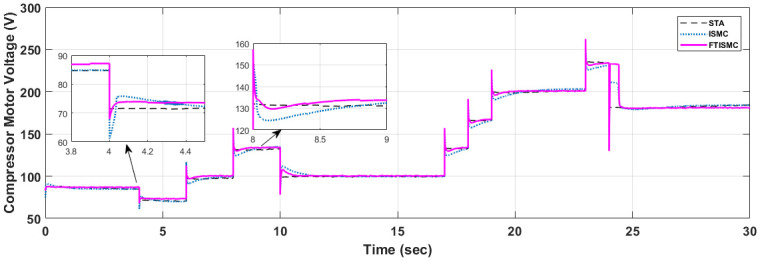
Compressor motor voltage.

The compressor motor controls the air supply that is being fed to the fuel cell. By controlling the compressor speed, it can be ensured that a balanced amount of oxygen is being provided to the system, which in turn promises smooth power delivery. The compressor speed is directly controlled using a compressor motor voltage signal which is shown in Fig 11. Comparing both the curves, we can conclude that FTISMC based strategy has spikes against transitions in current demands, so in this case, STA ensures the increased life span of the fuel cell’s compressor motor; however, as STA is a model less controller and it ignores all the compressor dynamics while all the compressor parameters have been considered when implementing ISMC and FTISMC so it might not be fair to compare the results of compressor motor voltage for STA with the variants of SMC in the presence of matched uncertainties for various current demand transitions. However, if we compare ISMC and FTISMC, ISMC gives an overdamped response, whereas FTISMC gives critically damped response. For example, at t = 10 s, the settling time for FTISMC is 1 s, and for ISMC, it is 1.5 s approximately.

**Fig 12:**
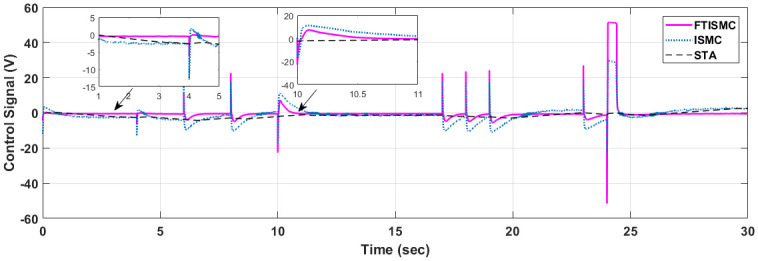
Control input.

The control gesticulations produced by both the FTISMC and ISMC control laws have been plotted for comparison in Fig 12. This figure shows that as compared to FTISMC, the control signal of the ISMC controller has greater magnitude, and at various time instants, there is a significant overshoot. For example, at t = 24 s, the control effort for FTISMC is maximum with the value of 50. However, the higher control effort results in almost zero error with the fastest settling time between the desired and actual compressor motor speed signal. As already discussed in the graphs shown in Fig 10. Similarly, if we compare the results at disturbance where t = 10 s, the strength of ISMC is approximately 11, whereas, at the same time instance, its value is 8 for FTISMC. So it can be concluded that when using ISMC the control effort is increased, which will be required to converge the error to zero more quickly than FTISMC. FTISMC gives better error tracking with lesser control effort as compared to ISMC. Moreover, some chattering can also be observed in the curve of ISMC. For example, from t = 12—17 s, the chattering against zero-crossing can be observed with a variation of 1 percent. OER when maintained at a specified level by applying the FTISMC and ISMC control techniques will ensure maximum efficiency and enhanced life span of the fuel cell by avoiding oxygen starvation or saturation.

## 6 Conclusion

FTISMC and ISMC control techniques have been presented in this article to regulate the air delivery of PEMFC. A performance-based comparative analysis of the above-mentioned techniques and STA is performed in this article. All three controllers were tested in the presence of matched uncertainties that have been added in compressor motor voltage, compressor speed, and supply manifold pressure. The performance comparison among three techniques proves that FTISMC outperforms ISMC and STA in the presence of matched uncertainties. The simulation results with the sudden variations in the load current requirements indicate that FTISMC exhibits improved response as compared to ISMC. FTISMC results have fewer percentage overshoots and faster settling time. FTISMC also overcomes the chattering phenomenon associated with conventional SMC and thus provides a jitter-free control signal.

The future scope will be to apply observer-based optimization techniques for designing the optimal control parameters for immeasurable states, resulting in further improvement of performance parameters. Moreover, a non-linear technique may be applied to control the hydrogen excess ratio for further performance enhancement.

## Supporting information

S1 Appendix (PDF)
