# Supplementary material for: Performance improvement in polymer electrolytic membrane fuel cell based on nonlinear control strategies—A comprehensive study
Source: PLoS One. 2022 Feb 25;17(2):e0264205. doi: 10.1371/journal.pone.0264205 (PMC8880434; doi:10.1371/journal.pone.0264205)
Supplement: S1 Appendix — (PDF) [file pone.0264205.s001.pdf]

Appendix

Abbreviations

Table 1. Abbreviations used in PEMFC mathematical model description.

C Concentration	D Diffusion coefficient (m^2/s)
F Faradays constant ($96485Cmol^{-1}$)	E Nernst voltage of a PEMFC (V)
M Molar mass (g/mol)	P Pressure (Pa)
i Stack current density (A/m^2)	I Current (A)
J Inertia (kg/m^2)	γ Specific heat ratio ($J/(kg \cdot K)$)
K Restriction constant	n Number of fuel cells in stack
λ Water content	

These are the main abbreviations of nomenclatures.

Table 2. Subscripts used in PEMFC mathematical model description.

an Anode	ca Cathode
w Water	a Air
H_2 Hydrogen	O_2 Oxygen
m Memberane	st Stack
cp Compressor motor	out Output / Exiting
hum Humidifier air	v Water vapour
net Total	$react$ Consumed in reaction
rm Return manifold	amb Ambient
N_2 Nitrogen	sat Saturation
sm Supply manifold	in Entering / input
ref Reference	

These are used as the subscripts of the main nomenclatures.
